# The Effectiveness of an Exercise Program on Muscle Strength and Range of Motion on Upper Limbs, Functional Ability and Depression at Early Stage of Dementia

**DOI:** 10.3390/jcm13144136

**Published:** 2024-07-15

**Authors:** Panagiotis Papamichail, Maria-Louiza Sagredaki, Christina Bouzineki, Sophia Kanellopoulou, Epameinondas Lyros, Anna Christakou

**Affiliations:** 1 Department of Physiotherapy, School of Health Sciences, University of Peloponnese, 23100 Sparta, Greece; p.papamic@gmail.com (P.P.); marialouizas@gmail.com (M.-L.S.); lyrosep@yahoo.gr (E.L.); 2 Intensive Care Unit, Bioclinic Athens, 11525 Athens, Greece; 3 Alzheimer Athens Association, 11636 Athens, Greece; cbouzineki@yahoo.com (C.B.); sophia.kanellopoulou@gmail.com (S.K.); 4 Department of Physiotherapy, Lab Biomechanics, School of Health Sciences, University of Peloponnese, 23100 Sparta, Greece; 5 Department of Physiotherapy, University of West Attica, 12243 Athens, Greece

**Keywords:** dementia, Alzheimer, exercise, functional ability, muscle strength, range of motion, depression

## Abstract

**Background**: Dementia involves the loss of cognitive abilities and represents a decline from the prior level of function, which impairs functional abilities in day-to-day life. The purpose of the present study is to examine the effectiveness of an exercise program on the muscle strength and range of motion of the upper limbs, the functional status, and the depression of elderly people with early stage dementia. **Methods**: The sample consisted of 60 elderly people with early stage dementia who were randomly divided into a control and an experimental group of 30 participants each. The experimental group received a 12-week Otago exercise program with 45 min duration of each session. The control group received usual care without doing exercise. The outcome measures of muscle strength and range of motion of the upper limbs, the functional status, and the depression were assessed by valid instruments and tests at the beginning and at the end of the intervention program. Repeated measures one-way ANOVA and Mann–Whitney tests examined the differences between the two groups at the end of the 12-week exercise program. **Results**: Statistically significant differences were found between the experimental and control groups in the entire outcome measures (functional ability F = 9.35 *p* < 0.05; muscle strength right hand F = 32.33, *p* < 0.05 left hand U = 95.50 *p* < 0.01; e.g., range of motion shoulder extension U = 104.00 *p* < 0.01), except depression. **Conclusions**: Both muscle strength and range of motion of the upper limbs, as well as the functional ability, were improved by the exercise program. Further research is needed to investigate the present results, in particular to explore the long-term cognitive, behavioral, and functional status outcomes of exercise in the early stages of dementia.

## 1. Introduction

Dementia is most commonly attributed to Alzheimer’s disease (AD), which causes impairment in areas of mental functioning such as memory, reasoning, concentration, and language and thus leads to poor quality of life, reduces functional ability, and increases mortality [1,2]. People with early stage dementia are characterized by loss of cognitive abilities (i.e., forgetting appointments, difficulty making decisions, being repetitive, misplacement of items), functional impairment in everyday life activities, and emotional symptoms (anxiety, depression, and lack of motivation). In the moderate stage of dementia, individuals cannot perform daily activities (i.e., cooking and washing). There is stiffness, loss of mobility, muscle atrophy, loss of muscle elasticity, and inability to perform skilled movements [3]. The above mobility problems ultimately lead to a loss of functional ability and self-care of the individual [4]. Also, they show a greater lack of orientation and memory problems and they become more intense. In the advanced stage of the disease, individuals experience both mobility problems and problems with feeding and urination, since they are not independently mobile. At this stage, the physical symptoms are even more apparent. Memory problems become more and more severe and patients find it difficult to recognize their friends and relatives as well as various objects [5,6].

Dealing with the possible aforementioned symptoms creates the need to include elderly people with dementia in exercise programs. Physical exercise, both aerobic and non-aerobic, is the only effective non-pharmacological intervention that has been shown to improve the functional ability of elderly people with AD [7,8,9]. The effect of exercise programs on elderly people with dementia is multifaceted, with improvements in lower limb muscle strength and muscle mass, the performance of the everyday daily activities, the ability of static and dynamic balance, the quality of life, and the psychological state, i.e., anxiety and depression. Τhe data on depression is controversial regarding its improvement [10]. Studies examined the effectiveness of the Otago Exercise Program (OEP) in elderly people with dementia and have shown that balance, functional ability, and quality of life are improved [11,12]. The Otago exercise program is the economical and practice program for elderly people. The OEP is comprised of strength and balance exercises. The OEP is recommended for older adults who are independent and can safely exercise alone or with a professional instructor. The program is particularly effective in individuals aged 80 years and older and is considered safe, effective, practical, and cost-effective approach to falls prevention [13,14,15]. Still, there are modified forms of the OEP that improve functionality and reduce falls.

There has been little research evaluating the effectiveness of an exercise program on the muscle strength and range of motion of the upper limbs in relation to the functional ability and the depression of people with early stage of dementia. Τhere are no studies assessing upper extremity range of motion in people with early stage dementia. There has been little research on the effect of exercise programs on upper extremity muscle strength [16]. In particular, Su et al. [16] found that the greater the cognitive decline, the lower the muscle strength of the upper limbs. Other studies have reported that there is an improvement in the functional ability of elderly people with early stage dementia [17,18]. Also, scarce research data are available on depression [10,19,20]. Νo study reported improvements in depression except the study of Cancela et al. [17].

The strength and endurance of upper limbs is crucial for elderly people due to their use in everyday activity. Therefore, the purpose of the study is to examine the effectiveness of the Otago exercise program on the muscle strength and range of motion of the upper limbs, functional status, and depression of people with early stage of dementia. The results of the present study may contribute to enhancing the rehabilitation programs. It was hypothesized that the experimental group who received the exercise program would have better muscle strength and range of motion of the upper limbs, higher level of functional ability, and less depression than the control group.

## 2. Materials and Methods

### 2.1. Study Design

This was a prospective, interventional experimental study with pre and post measures. This study has been registered and approved by the Ethics Committee of the School of Health Studies of the University of Peloponnese (203/06.04.2023) and the Scientific Council of the Alzheimer Athens Association (77A/06.04.2023). The study was in agreement with the Declaration of Helsinki Ethics principles. The participants received an allocation 1:1 to either a supervised 3-month Otago exercise program (Exercise Group) or not (Control Group) after a referral from the neurologist. Patients who could participate in the supervised Otago exercise program comprised the exercise group whereas patients who were unable to participate (due to transport issues) comprised the control group. A first baseline assessment prior to the intervention period (pre) and at the end of the post-assessment (post) was conducted by the end of the intervention period.

### 2.2. Sample

An a priori power analysis was conducted using G*Power version 3.1.9.7, which showed that we would need at least 21 participants per group in an independent samples *t*-test with an 80% power for detecting a large effect and a significance criterion of α = 0.05. Thus, 60 elderly people (37 women, 23 men) with average age 80 ± 6 years old were recruited from the Day Care Centre of the Alzheimer Association (Table 1). The inclusion criteria were as follows: (a) diagnosed early stage Alzheimer’s type dementia, (b) community-dwelling participants, (c) aged 65 to 95 years, (d) men and women, (e) good verbal and written communication and ability to follow instructions, (f) ability to walk, (g) no acute health problems in the last month (i.e., heart or respiratory failure, stroke), and (h) willingness to take part in the study. The exclusion criteria were as follows: (a) moderate- and severe-stage dementia, (b) severe psychiatric problems including psychosis, (c) serious health issues (i.e., medically significant cardiac or respiratory disease), and (d) inability to walk. The selection of individuals was carried out following a diagnosis by a neurologist with clinical tests and objective examinations. Specifically, diagnosis followed DSM-IV and NINCDS/ADRDA criteria [21]. The diagnostic tests applied by the neurologist once at the beginning for the inclusion of the participants in the program were as follows:

(a) Modified Addenbrooke’s Cognitive Examination- Revised (ACE-R) [22]
(b) A Frontal Assessment Battery [23], (c) Mini Mental State Examination (MMSE) score greater than or equal to 18 for incipient dementia, (d) Clock Drawing Test (e) The Short Anxiety Screening Test (SAST).

A signed consent form was completed by all participants after being informed about the procedure of the study. They were asked to complete an information sheet with the number of falls during the last year and their lifestyle and exercise habits. Demographic variables, including age, gender, occupation, and level of education, were considered. Their smoking and drinking status, medical problems, and medication were obtained from the information sheet. 

After registering, they were randomly divided into the following two groups by the method of drawing lots: (a) intervention group (exercise program), (b) control group (standard care). The *t*-tests for independent samples showed no statistical significance differences between the two groups (Table 1) and between the two genders in each group (Table 2) regarding baseline demographic data, as well as the clock drawing test and mini mental state examination performances. The enrollment and the assignment of participants to the allocation group were carried out by one of the authors. The recruitment of elderly people began in April 2023 and ended in July 2023. The experimental group followed the 3-month intervention program. The control group did not participate in any structured exercise program, but were, however, encouraged to engage in physical activity according to standard care. 

### 2.3. Materials

The outcome measures were as follows:

1. Upper extremity muscle strength
  - The Muscle Research Council assesses muscle strength in muscle groups. The six muscle groups assessed are the abductors of the humerus, flexors of the forearm, extensors of the wrist, hip flexors, knee flexors, and dorsiflexors of the ankle. The score ranges from 0–5, depending on the individual’s ability. The scale has good reliability in neurological diseases [24] but has not been used so far in neurodegenerative diseases (e.g., dementia).
  - The digital hand dynamometer is a tool for measuring upper extremity muscle strength. The hand dynamometer has very good reliability in people with early stage dementia (ICC = 0.97). The dimensions of the dynamometer (Charder Company, Taichung City, Taiwan, Model MG4800) are 100 mm × 212 mm × 55 mm; it weighs 400 g and is battery-operated [25].
2. Upper extremity range of motion

The goniometer is a tool for measuring the range of motion of joints in the human body. The hand goniometer has not been used so far to assess people with early stage dementia but has been widely used for upper extremity pathologies of the musculoskeletal system (Meloq Company, Stockholm, Sweden, Model Easy Angle Goniometer) [26].

3. Functional status

The Timed Up and Go test (TUG) is a test used to examine a person’s mobility. It assesses the time it takes a participant to rise from a chair, walk three meters, turn around 180 degrees, walk back to the chair, and sit down while turning 180 degrees [27]. As soon as the patient is seated, the timing stops. The time required to perform the test is approximately 10–35 s. The test has good reliability in patients with Alzheimer’s disease [28].

4. Depression

The Geriatric Depression Scale initially had 30 questions, while its short form used in this research consists of 15 questions, of which 10 show the presence of depression when answered positively, while the rest of the questions show depression when answered negatively. A score of 0–4 is normal, 5–8 indicates mild depression, 9–11 indicates moderate depression, and 12–15 indicates severe depression. The time to complete it ranges from 5–7 min. The scale has 92% sensitivity and is reliable and valid with a high correlation index (r = 0.84, *p* < 0.001) [29]^,^ and there is a version in Greek available [30].

### 2.4. Procedure

The intervention study was carried out in a private clinical setting by a team of physiotherapists. Initially, information was given on the purpose, importance, and process of the study. The participants carried out the Otago exercise program under the supervision of the same experienced trained physiotherapist at the Day Care Centre of the Alzheimer Association. They received 24 physiotherapy sessions of Otago exercise program, lasting 45 min each, twice a week. The duration of the physiotherapy program was three months (12 weeks). At first, we conducted a pilot study with two participants to test the protocol of the intervention program, and afterwards, the study started. 

The physiotherapy exercise program included exercises selected from the Otago Exercise Program (OEP) which was developed and tested by the New Zealand Falls Prevention Research Group in New Zealand to reduce falls in older persons. Yet little research has investigated the use of the OEP in people with dementia [12]. The exercise program was of moderate intensity and included exercises from the Otago Exercise Program. The program included a warm-up, the main part, and recovery exercises. The main part consisted of muscle strengthening and balance exercises for the upper and lower limbs. In particular, the OEP consisted of a warm-up stage promoting circulation and preparing the body for the rest of the program. Participants mobilized their joints and stretched their muscles. Strength exercises programs, i.e., resistance training protocols with the use of weights, can improve muscle strength, physical performance, and endurance in elderly people [31]. Balance is essential to improve posture and perform everyday activities. Dynamic and static balance exercises may also increase confidence and reduce the possibility of a fall. Finally, stretching exercises develop flexibility and promote relaxation. They reduce the likelihood of fatigue and revitalize the body at the end of an exercise session. 

The exercise program may include the following: (1) easy marching, (2) head movements, (3) back extensions, (4) ankle movements, (5) front and back knee strengthening, (6) slide hip strengthening, (7) calf and toe raises hold, (8) toe and heel walking, (9) one leg stances, (10) sideways walking, (11) sit to stand, and (12) back of thigh and calf stretches. All participants performed identical exercises during their program. They were advanced to the next level of exercises according to the Otago exercise protocol. The participants were assessed before the start and at the end of the exercise program. The initial and final assessment of the participants was carried out by a different author, without the author knowing which group the participant belonged to.

## 3. Results

Descriptive statistics examined the demographical data of the sample (Table 1). A normality test of the distribution of all variables was performed using the Kolmogorov–Smirnov test. Repeated measures one-way ANOVA and Mann–Whitney tests examined the differences between the two groups at the end of the 12 weeks exercise program. Apart from depression, all variables improved post-exercise in favor of the experimental group. In particular, the results revealed statistically significant differences in (a) functional status (Table 3), (b) handgrip strength for the right upper limb (Table 3) and for the left upper limb (Table 4), (c) muscle strength (Table 5), and (d) range of motion (Table 6) of the upper limbs between the experimental and the control group. No statistically differences appeared in the depression variable between the two groups.

## 4. Discussion

The present study investigated the effectiveness of an exercise program on muscle strength and range of motion of upper limbs, functional status, and depression in elderly people with early stages of dementia. The results showed that the 12-week exercise program can benefit muscle strength and range of motion of both upper limbs, as well as functional status, of elderly people with mild dementia. The only muscle groups that showed less improvement in the exercise group were the ulnar/radial deviators and the abductors, possibly due to participants’ other musculoskeletal and geriatric conditions affecting these muscles [32]. The greater improvement in the exercise group’s hand grip strength is attributed to the hand’s isometric exercises. In agreement with our results, [26] showed improvement for hand grip strength.

Another study found that the greater the cognitive decrease, the lower the strength of the upper limbs of patients with early AD and mild cognitive impairment. That means that healthy participants had better hand grip strength compared to those with mild cognitive impairment and AD. Among them, participants with AD had reduced muscle strength compared to those with mild cognitive impairment [32]. Hatabe et al. [33] found a greater decline in hand grip strength in individuals with greater cognitive impairment. Reduced hand grip strength may be a predictor for developing dementia [34]. Similar results were found by another study [35] between hand grip strength and cognitive function. The authors suggested that hand grip strength might be a useful variable to assess the course of the disease, as well as the individual’s cognitive status [35]. Therefore, hand grip strength is suggested as a reliable monitoring tool of cognitive decline. Studies highlight that the relationship of hand grip strength and cognitive function is due to overlapping domains of motor coordination and cognitive function [36,37]. More research should be conducted to confirm these results.

There is no previous research examining upper extremities’ range of motion of elderly people with dementia. Upper extremity range of motion has been studied in populations with other neurodegenerative diseases such as Parkinson’s disease and multiple sclerosis [38,39,40]. Particularly, in a Parkinson’s disease study, the therapeutic exercise program appeared to increase patients’ range of motion after an 8-week exercise intervention [41]. In the present study, the range of motion of the upper limbs showed significant differences for most of the joints of both upper extremities in the experimental group compared to the control group. Specifically, in the right upper extremity, significant differences were observed in extension, abduction, adduction, internal and external rotation of the shoulder, flexion, extension, pronation, and supination of the elbow and in flexion, extension, ulnar and radial deviation of the wrist. Regarding shoulder flexion, there is a tendency for the experimental group to improve over the control group, without this being confirmed by statistical analyses. In the left upper extremity, significant differences were found in extension, internal and external rotation of the shoulder, flexion, extension, pronation and supination of the elbow, and extension, radial deviation and ulnar deviation of the wrist. Regarding shoulder flexion, abduction, and adduction, as well as for wrist flexion, the experimental group was more improved over the control group but the results did not reach statistical significance. Elderly people had arthritis of different forms such as osteoarthritis or deforming arthritis, thus reducing their range of motion. Exercise reduces swelling, inflammation, pain, and joint stiffness in elderly people and contributes to increasing their range of motion [42].

The exercise program appeared to improve the functional ability of elderly people with early stage dementia. The increase in upper limb strength and range of motion resulted in increased upper limb swing during gait. This has the effect of increasing walking speed and boosting self-confidence of these individuals in performing daily activities. Similar to the present study’s results, other study showed that functional ability showed an improvement in the group following an exercise program compared to the control group, but without a statistical difference [17]. Also, Blankevoort et al. [43] reported that combined exercise programs including endurance, strength, and balance exercises improve functional ability in elderly people with dementia regardless of its stage. Similarly Cezar et al. [18] implemented a remotely supervised exercise program in a sample consisting of 40 participants with early to moderate dementia, while the present study consisted of 60 participants with early stage dementia.

However, the elderly people in the exercise group did not experience a reduction in depression symptoms, possibly because the majority of elderly people appeared to be free of depression anyway. Another possible explanation may be that the depression questionnaire did not reflect any small clinical changes in the participants’ depression after the exercise program. Other studies have shown similar results and failed to show significant improvements in depression levels of elderly people with dementia who followed an exercise program [19,21]. On the contrary, another study showed some significant benefits of exercise in elderly people with dementia who were depressed after following an exercise program compared to a control group [44]. Another study showed increased depression in the experimental group and lower depression in the control group, without specifying whether the study sample included patients with different stages of dementia, such as early or moderate stage [17].

The present study has some limitations. The sample consisted of participants with early onset dementia, and the results cannot be generalized to other stages of dementia or other neurodegenerative diseases. An additional limitation lies in the use of the depression assessment instrument based on self-evaluations of patients that probably failed to detect any small clinical changes. Due to the lack of studies, it is necessary to analyze, in depth, the effect of exercise programs on the range of motion of elderly people with dementia and how this changes with the progression of the disease. Future studies might examine the relationship between cognitive status and upper extremity muscle strength before and after the implementation of an exercise program using larger samples and while examining other physical and psychological variables like neuromuscular coordination, self-efficacy, and confidence. Also, future research should be conducted to assess gender differences after the intervention program in all the examined dependent variables of the study.

## 5. Conclusions

The exercise program can improve the muscle strength and range of motion of the upper limbs, as well as the functional ability, of elderly people with early stage Alzheimer’s dementia. However, exercise did not appear to reduce depression. Further research is necessary to explore the results of the present study, using a larger sample size, adopting a different approach of assessing depression, and evaluating whether any effects persist over time after the end of the exercise program.

## Figures and Tables

**Table 1:** Baseline demographic and clinical-cognitive characteristics between the experimental and control group.

Variables	Experimental Group (n = 30)	Control Group (n = 30)	t
Sex, n (%)	Male 10 (33.3) Female 20 (66.6)	Male 13 (43.3) Female 17 (56.7)	
Age (y), mean ± SD	80.17 ± 6.37	79.83 ± 5.83	0.21
Clock Drawing Test, mean ± SD	12.20 ± 2.41	12.67 ± 0.92	0.98
Mini Mental State Examination, mean ± SD	21.50 ± 1.79	22.00 ± 2.13	0.99

**Table 2:** Baseline demographic and clinical-cognitive characteristics between males and females of the experimental and control group.

	Experimental Group			Control Group		
Variables	Male (n = 10)	Female (n = 20)	t	Male (n = 13)	Female (n = 17)	t
Age (y), mean ± SD	81.00 ± 5.65	79.75 ± 6.80	0.5	80.58 ± 4.27	79.41 ± 6.89	0.28
Mini Mental State Examination, mean ± SD	21.70 ± 1.88	22.15 ± 2.27	0.53	22.69 ± 1.97	20.59 ± 0.93	3.54
Clock Drawing Test, mean ± SD	12.10 ± 0.56	12.95 ± 0.94	3.06	12.69 ± 1.37	11.88 ± 2.96	0.97
Body height (cm)	172.80 ± 2.57	157.65 ± 2.41	15.8	171.92 ± 2.06	158.65 ± 2.87	14.10
Body weight (kg)	79.90 ± 5.76	67.65 ± 6.49	5.04	74.62 ± 4.33	64.88 ± 4.51	5.95

**Table 3:** Differences in functional status and handgrip strength right hand between experimental and control group with one–way ANOVA test.

Experimental Group		Control Group		
Variables	Μ ± SD	Μ ± SD	F	df
Functional status (sec)	9.21 ± 2.08	10.91 ± 2.22	9.35 *	1.58
Handgrip strength right hand (kg)	18.62 ± 5.77	11.63 ± 3.65	31.33 *	

* *p* < 0.05

**Table 4:** Differences in handgrip strength left hand between the experimental and control group.

Groups	Handgrip Strength Left Hand (kg)		
	M rank	U	Z
Experimental group	42.32	95.50 **	5.24
Control group	18.68		

** *p* < 0.001.

**Table 5:** Differences in muscular strength by Medical Research Council (MRC, total score) scale and Geriatric Scale Depression (GDS) between experimental and control group.

		Experimental Group	Control Group	Mann–Whitney U	
Variables	Arm	M rank	M rank	U	Z
MRC shoulders abductions	Right	36.02	24.98	284.50 *	1.19
MRC forearm flexors	Right	39.67	21.33	175.00 **	4.27
MRC forearm extensors	Right	40.25	20.75	157.50 **	4.61
MRC forearm pronators	Right	38.85	21.15	169.50 **	4.42
MRC forearm supinators	Right	36.70	24.30	264.00 *	2.88
MRC wrists extensors	Right	36.93	24.07	257.00 *	3.04
MRC wrists flexors	Right	37.05	23.95	253.50 **	3.19
MRC shoulders abductors	Left	36.15	24.85	280.50 *	1.56
MRC forearm flexors	Left	38.23	22.77	218.00 **	3.64
MRC forearm extensors	Left	38.23	22.77	218.00 **	3.64
MRC forearm pronators	Left	37.32	23.68	245.50 **	3.28
MRC forearm supinators	Left	37.48	23.52	240.00 **	3.22
MRC wrists extensors	Left	37.10	23.90	252.00 **	3.23
MRC wrists flexors	Left	37.50	23.50	240.00 **	3.32
MRC wrists radial deviators	Left	36.48	24.52	270.50 **	3.10
Geriatric Scale Depression (GDS)	-	30.98	30.02	435.00	0.38

* *p* < 0.05, ** *p* < 0.001.

**Table 6:** Differences in range of motion (in degrees) between experimental and control group.

Variables		Experimental Group	Control Group	Mann–Whitney U	
	Arm	M rank	M rank	U	Z
ROM of shoulder extension	Right	42.03	18.97	104.00 **	5.14
ROM shoulder abduction	Right	22.38	38.62	206.50 **	3.6
ROM shoulders internal rotation	Right	37.68	23.32	234.50 *	3.18
ROM shoulders external rotation	Right	42.83	18.17	80.00 **	5.51
ROM elbows extension	Right	35.60	25.40	297.00 *	3.16
ROM elbows flexion	Right	37.60	23.40	237.00 **	2.26
ROM elbows pronation	Right	40.22	20.78	158.50 **	4.32
ROM elbows suprination	Right	35.52	25.48	299.50 *	2.22
ROM wrists flexion	Right	25.48	32.52	299.50 *	2.22
ROM wrists extension	Right	41.75	19.25	112.50 **	5.00
ROM wrists radial deviation	Right	39.97	21.03	112.50 **	4.21
ROM of shoulder extension	Left	43.70	17.30	54.00 **	5.86
ROM shoulders internal rotation	Left	39.98	22.02	195.50 **	3.76
ROM elbows flexion	Left	37.88	23.12	228.50 *	3.27
ROM elbows extension	Left	38.15	22.85	220.50 *	3.42
ROM elbows pronation	Left	40.58	20.42	147.50 **	4.47
ROM elbows suprination	Left	37.92	23.08	227.50 *	3.29
ROM wrists extension	Left	41.18	18.82	129.50 **	2.74
ROM wrists radial deviation	Left	41.55	19.45	118.50 **	4.91

* *p* < 0.05, ** *p* < 0.001.

## Data Availability

Data available on request from the corresponding author.
